# Notification of changes in taxonomic opinion previously published outside the IJSEM. List of changes in taxonomic opinion no. 40

**DOI:** 10.1099/ijsem.0.006482

**Published:** 2024-08-13

**Authors:** Aharon Oren, Markus Göker

**Affiliations:** 1 The Institute of Life Sciences, The Hebrew University of Jerusalem, The Edmond J. Safra Campus, 9190401 Jerusalem, Israel; 2 Leibniz Institute DSMZ – German Collection of Microorganisms and Cell Cultures, Inhoffenstrasse 7B, 38124 Braunschweig, Germany

The International Code of Nomenclature of Prokaryotes (ICNP) [1] deals with the nomenclature of prokaryotes. This may include existing names (the Approved Lists of Bacterial Names) as well as new names and new combinations. In this sense, the Code is also dealing indirectly with taxonomic opinions. However, as with most codes of nomenclature, there are no mechanisms for formally recording taxonomic opinions that do not involve the creation of new names or new combinations. In particular, it would be desirable for taxonomic opinions resulting from the creation of synonyms or emended descriptions to be made widely available to the public. In 2004, the Editorial Board of the *International Journal of Systematic and Evolutionary Microbiology* (IJSEM) agreed unanimously that it was desirable to cover such changes in taxonomic opinions (i.e. the creation of synonyms or the emendation of circumscriptions) previously published outside the IJSEM, and to introduce a List of Changes in Taxonomic Opinion [2]. Scientists wishing to have changes in taxonomic opinion included in future lists should send a PDF file of the relevant publication to the IJSEM Editorial Office or to the List Editors.

It must be stressed that the date of proposed taxonomic changes is the date of the original publication, not the date of publication of the list. Taxonomic opinions included in the List of Changes in Taxonomic Opinion do not affect the status of a name under the ICNP [2] and can neither by considered as approved nor as disapproved by the International Committee on Systematics of Prokaryotes. The names that are to be used are those that are the ‘correct names’ (in the sense of Principle 6 of the ICNP) in the opinion of the microbiologist, with a given circumscription, position and rank. A particular name, circumscription, position and rank does not have to be adopted in all circumstances. Consequently, the List of Changes in Taxonomic Opinion must be considered as a service to microbiology and it has no ‘official character’, other than providing a centralized point for registering/indexing such changes in a way that makes them easily accessible to the scientific community.

The List Editors reserve the right not to include in the list proposed emendations that are in our opinion unimportant and do not significantly alter the original description of the taxon. This includes addition of new members to the taxon, reclassification of members in a different taxon, addition of genome sequence data, and other changes that marginally change the taxon circumscription [3]. Conversely, the mere inclusion of an emendation in a List of Changes in Taxonomic Opinion does not indicate that the List Editors regard this emendation as taxonomically relevant.

**Table:** 

Name/authors	Proposed as	Reference
*Aedoeadaptatus* Hitch *et al*. 2022 emend. Malhotra *et al*. 2024, 13*	emend.	[4]
*Chromohalobacter salexigens* Arahal *et al*. 2001 pro synon. *Chromohalobacter israelensis* (Huval *et al*. 1996) Arahal *et al*. 2001	synon.	[5]
*Cobetia amphilecti* Romanenko *et al*. 2013 emend. Nedashkovskaya *et al*. 2024, 13*	emend.	[6]
*Cobetia litoralis* Romanenko *et al*. 2013 pro synon. *Cobetia amphilecti* Romanenko *et al*. 2013	synon.	[6]
*Cobetia marina* (Cobet *et al*. 1970) Arahal *et al*. 2002 emend. Nedashkovskaya *et al*. 2024, 13*	emend.	[6]
*Cobetia pacifica* Romanenko *et al*. 2013 pro synon. *Cobetia marina* (Cobet *et al*. 1970) Arahal *et al*. 2002	synon.	[6]
*Corynebacteriineae* corrig. Stackebrandt *et al*. 1997 emend. Val-Calvo and Vázquez-Boland 2023, 16*	emend.	[7]
*Curtobacterium albidum* (Komagata and Iizuka 1964) Yamada and Komagata 1972 (Approved Lists 1980) pro synon. *Curtobacterium citreum* (Komagata and Iizuka 1964) Yamada and Komagata 1972 (Approved Lists 1980)	synon.	[8]
*Curtobacterium citreum* (Komagata and Iizuka 1964) Yamada and Komagata 1972 (Approved Lists 1980) emend. Osdaghi *et al*. 2024, 10*	emend.	[8]
*Curtobacterium flaccumfaciens* (Hedges 1922) Collins and Jones 1984 emend. Osdaghi *et al*. 2024, 10*	emend.	[8]
*Fusobacterium equinum* Dorsch *et al*. 2001 pro synon. *Fusobacterium gonidiaformans* (Tunnicliff and Jackson 1925) Moore and Holdeman 1970 (Approved Lists 1980)	synon.	[9]
*Fusobacterium necrophorum* subsp. *funduliforme* (*ex* Hallé 1898) Shinjo *et al*. 1991 pro synon. *Fusobacterium necrophorum* subsp. *necrophorum* (Flügge 1886) Shinjo *et al*. 1991	synon.	[9]
*Haloarcula* Torreblanca *et al*. 1986 emend. Ma *et al*. 2024, 8*	emend.	[10]
*Halobellus ramosii* Pérez-Davó *et al*. 2015 pro synon. *Halobellus inordinatus* Qiu *et al*. 2013	synon.	[11]
*Haloferax alexandrinus* Asker and Ohta 2002 pro synon. *Haloferax volcanii* (Mullakhanbhai and Larsen 1975) Torreblanca *et al*. 1986^‡^	synon.	[11]
*Haloferax lucentense* corrig. Gutierrez *et al*. 2004 pro synon. *Haloferax volcanii* (Mullakhanbhai and Larsen 1975) Torreblanca *et al*. 1986	synon.	[11]
*Halogranum salarium* Kim *et al*. 2012 pro synon. *Halogranum rubrum* Cui *et al*. 2010	synon.	[11]
*Halomonas alkaliantarctica* Poli *et al*. 2011 pro synon. *Halomonas neptunia* Kaye *et al*. 2004	synon.	[5]
*Halomonas axialensis* Kaye *et al*. 2004 pro synon. *Halomonas aquamarina* (ZoBell and Upham 1944) Dobson and Franzmann 1996	synon.	[5]
*Halomonas hydrothermalis* Kaye *et al.* 2004 pro synon. *Halomonas venusta* (Baumann *et al*. 1972) Dobson and Franzmann 1996	synon.	[5]
*Halomonas johnsoniae* Kim *et al.* 2010 pro synon. *Halomonas hamiltonii* Kim *et al.* 2010	synon.	[5]
*Halomonas meridiana *James *et al*. 1990 pro synon. *Halomonas aquamarina* (ZoBell and Upham 1944) Dobson and Franzmann 1996	synon.	[5]
*Halomonas salina* (Valderrama *et al*. 1991) Dobson and Franzmann 1996 pro synon. *Halomonas halophila* (Quesada *et al*. 1984) Dobson and Franzmann 1996	synon.	[5]
*Haloplanus rallus* Cho *et al*. 2018 pro synon. *Haloplanus salinarum* Hwang *et al*. 2017	synon.	[9]
*Halosegnis longus* Durán-Viseras *et al*. 2022 emend. Hu *et al*. 2024, 8^*^	emend.	[12]
*Jatrophihabitans* Madhaiyan *et al*. 2013 emend. Suh *et al*. 2024, 6^*^	emend.	[13]
*Litoribacter alkaliphilus* Subhash *et al*. 2013 pro synon. *Litoribacter ruber* Tian *et al*. 2010	synon.	[14]
*Microbacterium ketosireducens* Takeuchi and Hatano 1998 pro synon. *Microbacterium terrae* (Yokota *et al*. 1993) Takeuchi and Hatano 1998^II^	synon.	[15]
*Microbacterium kitamiense* Matsuyama *et al*. 1999 pro synon. *Microbacterium aurantiacum* Takeuchi and Hatano 1998^II^	synon.	[15]
*Microbacterium maritypicum* corrig. (ZoBell and Upham 1944) Takeuchi and Hatano 1998^II^ pro synon. *Microbacterium liquefaciens* (Collins *et al*. 1983 *ex* Orla-Jensen 1919) Takeuchi and Hatano 1998^II^	synon.	[15]
*Modicisalibacter* Ben Ali Gam *et al*. 2007 emend. de la Haba *et al*. 2023, 24	emend.	[5]
*Mycobacterium* Lehmann and Neumann 1896 (Approved Lists 1980) emend. Val-Calvo and Vázquez-Boland 2023, 16*	emend.	[7]
*Mycobacteroides* Gupta *et al*. 2018 emend. Val-Calvo and Vázquez-Boland 2023, 16*	emend.	[7]
*Paraglaciecola agarilytica* (Yong *et al*. 2007) Shivaji and Reddy 2014 emend. Emsley *et al*. 2024, 13*	emend.	[16]
*Paraglaciecola oceanifecundans* Shivaji and Reddy 2014 pro synon. *Paraglaciecola agarilytica* (Yong *et al*. 2007) Shivaji and Reddy 2014	synon.	[16]
*Peptoniphilus porci* Wylensek *et al*. 2021 pro synon. *Peptoniphilus faecalis* Ryu *et al*. 2021	synon.	[4]
*Pseudomonas alcaligenes* Monias 1928 (Approved Lists 1980) emend. Ling *et al*. 2023, 562^#^	emend.	[17]
*Rhodococcus* Zopf 1891 (Approved Lists 1980) emend. Val-Calvo and Vázquez-Boland 2023, 16*	emend.	[7]
*Sciscionella* Tian *et al*. 2009 emend. Teo *et al*. 2024, 6*	emend.	[18]
*Staphylococcus delphini* Varaldo *et al*. 1988 emend. Vrbovska *et al*. 2020, 13*	emend.	[19]
*Streptomyces demainii* Goodfellow *et al*. 2008 pro synon. *Streptomyces hygroscopicus* (Jensen 1931) Yüntsen *et al*. 1956 (Approved Lists 1980)	synon.	[20]
*Streptomyces nashvillensis* McVeigh and Reyes 1961 (Approved Lists 1980) pro synon. *Streptomyces tanashiensis* Hata *et al*. 1952 (Approved Lists 1980)	synon.	[21]
*Streptomyces sporocinereus* (*ex* Krassilnikov 1970) Preobrazhenskaya 1986 pro synon. *Streptomyces hygroscopicus* (Jensen 1931) Yüntsen *et al*. 1956 (Approved Lists 1980)	synon.	[20]
*Streptomyces tanashiensis* Hata *et al*. 1952 (Approved Lists 1980) emend. Ates *et al*. 2023, 1085	emend.	[21]
*Thiorhodovibrio* Overmann *et al*. 1993 emend. Methner *et al*. 2023, 16*	emend.	[22]
*Thiorhodovibrio winogradskyi* Overmann *et al*. 1993 emend. Methner *et al*. 2023, 16*	emend.	[22]

* The journal in which the name was effective published does not have continuous pages.

‡ *alexandrinus* (or grammatically correct *alexandrinum*) was misspelled *alexandrines*.

II This species is classified as Risk Group 2.

# The authors stated “Emended description of the strain *Pseudomonas alcaligenes* ATCC 14909T” instead of “Emended description of *Pseudomonas alcaligenes* Monias 1928 (Approved Lists 1980)”.

For references to Lists of Changes in Taxonomic Opinion no. 1–39 see *Int J Syst Evol Microbiol* **55** (2005) 7, 1403; **56** (2006) 11, 1463; **57** (2007) 4, 1376; **58** (2008) 5, 1515; **59** (2009) 5, 1559; **60** (2010) 5, 1482; **61** (2011) 4, 1504; **62** (2012) 7, 1448; **63** (2013), 8, 2371, **64** (2014), 8, 2191; **65** (2015), 7, 2028; **66** (2016), 7, 2469; **67** (2017), 7, 2081; **68** (2018), 7, 2137; **69** (2019), 13, 1850; **70** (2020), 9, 4061; **71** (2021), 004596, 004816; **72** (2022), 005164, 005413; **73** (2023), 005696, 005923; **74** (2024), 006145.
